# The chromosome-level genome sequences of *Danio rerio* strains AB, Nadia and Cooch Behar

**DOI:** 10.12688/wellcomeopenres.25012.1

**Published:** 2025-10-10

**Authors:** Kerstin Howe, Zoltan Varga, John Postlethwait, Shane A. McCarthy, Jonathan M. D. Wood, Michelle Smith, Karen Oliver

**Affiliations:** 1 Tree of Life programme, Wellcome Sanger Institute, Hinxton, England, UK; 2 Zebrafish International Resource Center (ZIRC), Eugene, Oregon, USA; 3 College of Arts and Sciences, University of Oregon, Eugene, Oregon, USA; 4 Wellcome Sanger Institute, Hinxton, England, UK

**Keywords:** Danio rerio AB strain, Nadia strain, Cooch Behar strain, zebrafish, genome sequence, chromosomal, Cypriniformes

## Abstract

We present the genome assemblies of three females of the *Danio rerio* strains, AB, Nadia and Cooch Behar (zebrafish; Chordata; Actinopteri; Cypriniformes; Cyprinidae). These assemblies were released in 2020 as part of the Danioninae Sequencing Project. The genome sequence of the strain AB is 1,405.10 megabases, the Nadia strain 1,465.10, and the Cooch Behar strain 1,421.80 megabases in length. Most of the assembly is scaffolded into 25 chromosomal pseudomolecules in each case. For each strain, the mitochondrial genome was also assembled and is 16.6 kilobases in length.

## Species taxonomy

Eukaryota; Opisthokonta; Metazoa; Eumetazoa; Bilateria; Deuterostomia; Chordata; Craniata; Vertebrata; Gnathostomata; Teleostomi; Euteleostomi; Actinopterygii; Actinopteri; Neopterygii; Teleostei; Osteoglossocephalai; Clupeocephala; Otomorpha; Ostariophysi; Otophysi; Cypriniphysae; Cypriniformes; Cyprinoidei; Danionidae; Danioninae; *Danio*; *Danio rerio* (Hamilton, 1822) (NCBI:txid7955).

## Background

*Danio rerio*, commonly known as the zebrafish, serves as a model organism in various fields of biological research, including genetics, developmental biology, and toxicology ( Lieschke & Currie, 2007). Native to the freshwater habitats of South Asia, the genome of this teleost fish was fully sequenced in 2013 ( Howe *et al.*, 2013). The genome has a span of around 1.4 gigabases, distributed across 25 chromosomes. Notably, the zebrafish genome exhibits a high degree of synteny with the human genome, making it a valuable tool for studying gene function and regulation ( Howe *et al.*, 2013; Postlethwait *et al.*, 2000).

One of the advantages of using *Danio rerio* as a model organism is its rapid embryonic development, which is largely transparent and easily observable under a microscope ( Kimmel *et al.*, 1995). This facilitates real-time analysis of developmental processes. Additionally, the zebrafish is amenable to genetic manipulation, including techniques such as CRISPR/Cas9, which allows for targeted gene editing ( Hwang *et al.*, 2013). Its relatively low maintenance cost and high fecundity further contribute to its utility in research settings ( Lawrence, 2007).

The availability of a fully sequenced genome has accelerated functional genomics studies in zebrafish, enabling researchers to perform genome-wide association studies (GWAS), transcriptomics, and other high-throughput analyses ( Lieschke & Currie, 2007). Consequently, *Danio rerio* continues to be an indispensable resource in advancing our understanding of vertebrate biology, genetics, and disease mechanisms.

In addition to its genomic attributes, the zebrafish model is further enriched by the diversity of its classical laboratory strains. Whilst the zebrafish reference genome assemblies have historically been based on sequencing the Tuebingen strain (TU; ZFIN ID: ZDB-GENO-990623–3), other strains have been widely used by the research community. We present here the genome assemblies for three zebrafish strains: AB (ZFIN ID: ZDB-GENO-960809–7), Nadia (NA; ZFIN ID: ZDB-GENO-030115-2) ( Anderson *et al.*, 2012) and Cooch Behar (CB) ( Wilson *et al.*, 2014). The AB strain functions as a long-established laboratory strain used in parallel to Tuebingen, whereas Nadia and Cooch Behar are relatively new, wild caught additions.

We present the genome assemblies of an individual of the *Danio rerio* AB strain based on a sample provided by Zoltan Varga at ZIRC, and of individuals of the *Danio rerio* Nadia and Cooch Behar strains based on samples provided by John Postlethwait, University of Oregon, via ZIRC.

## Genome sequence reports

**Figure 1.**
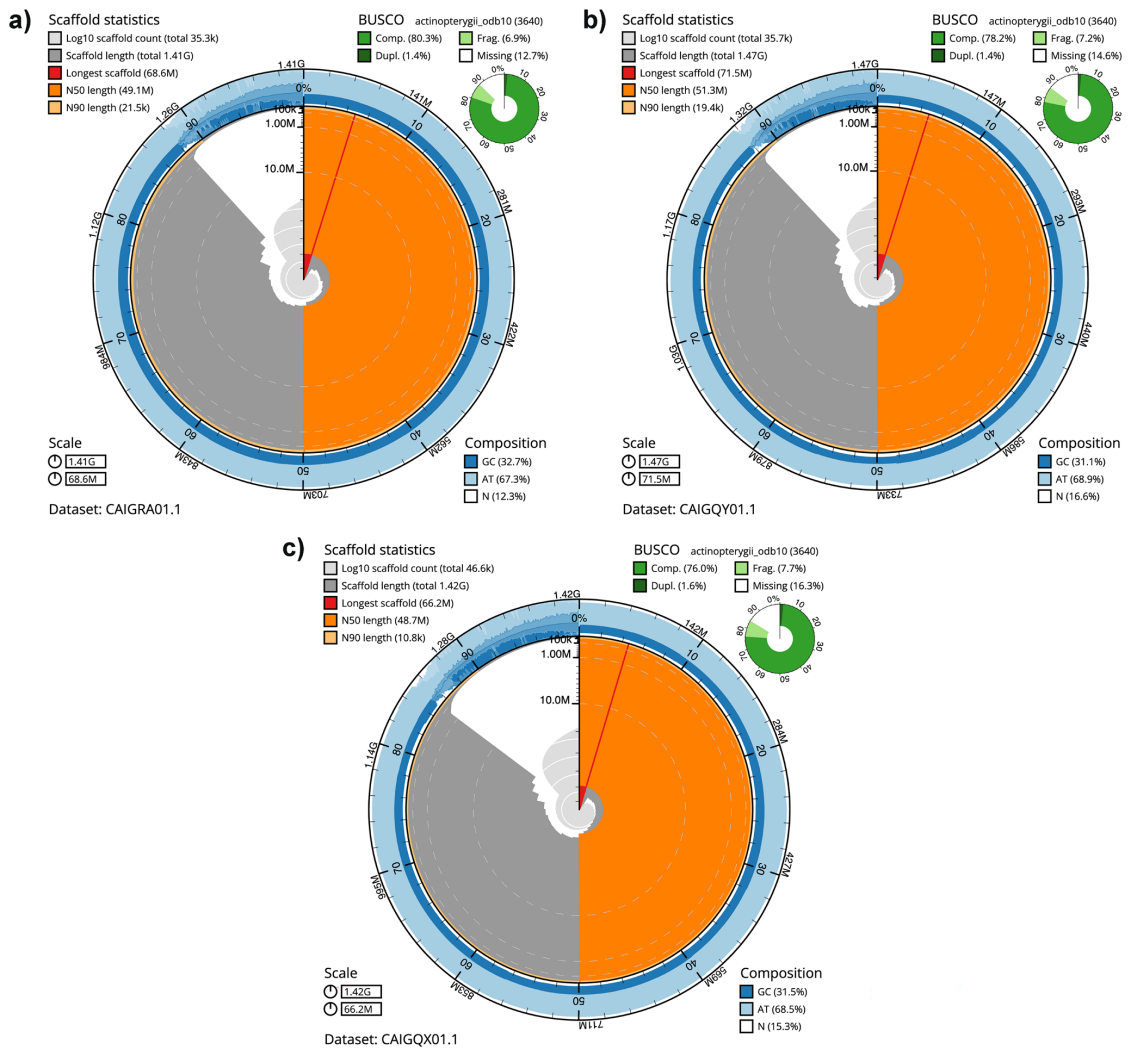
Genome assembly of *Danio rerio* strains: BlobToolKit snail plots for **a**) fDreABz2.1, **b**) fDreNAz3.1 and **c**) fDreCBz1.1. The BlobToolKit snail plots show N50 metrics and BUSCO gene completeness. The main plot is divided into 1,000 size-ordered bins around the circumference with each bin representing 0.1% of the assembly. The distribution of sequence lengths is shown in dark grey with the plot radius scaled to the longest sequence present in the assembly (shown in red). Orange and pale-orange arcs show the N50 and N90 sequence lengths, respectively. The pale grey spiral shows the cumulative sequence count on a log scale with white scale lines showing successive orders of magnitude. The blue and pale-blue area around the outside of the plot shows the distribution of GC, AT and N percentages in the same bins as the inner plot. A summary of complete, fragmented, duplicated and missing BUSCO genes in the actinopterygii_odb10 set is shown in the top right. The snail plots can be viewed interactively at the following links: fDreABz2, fDreNAz3 and fDreCBz1.

**Figure 2.**
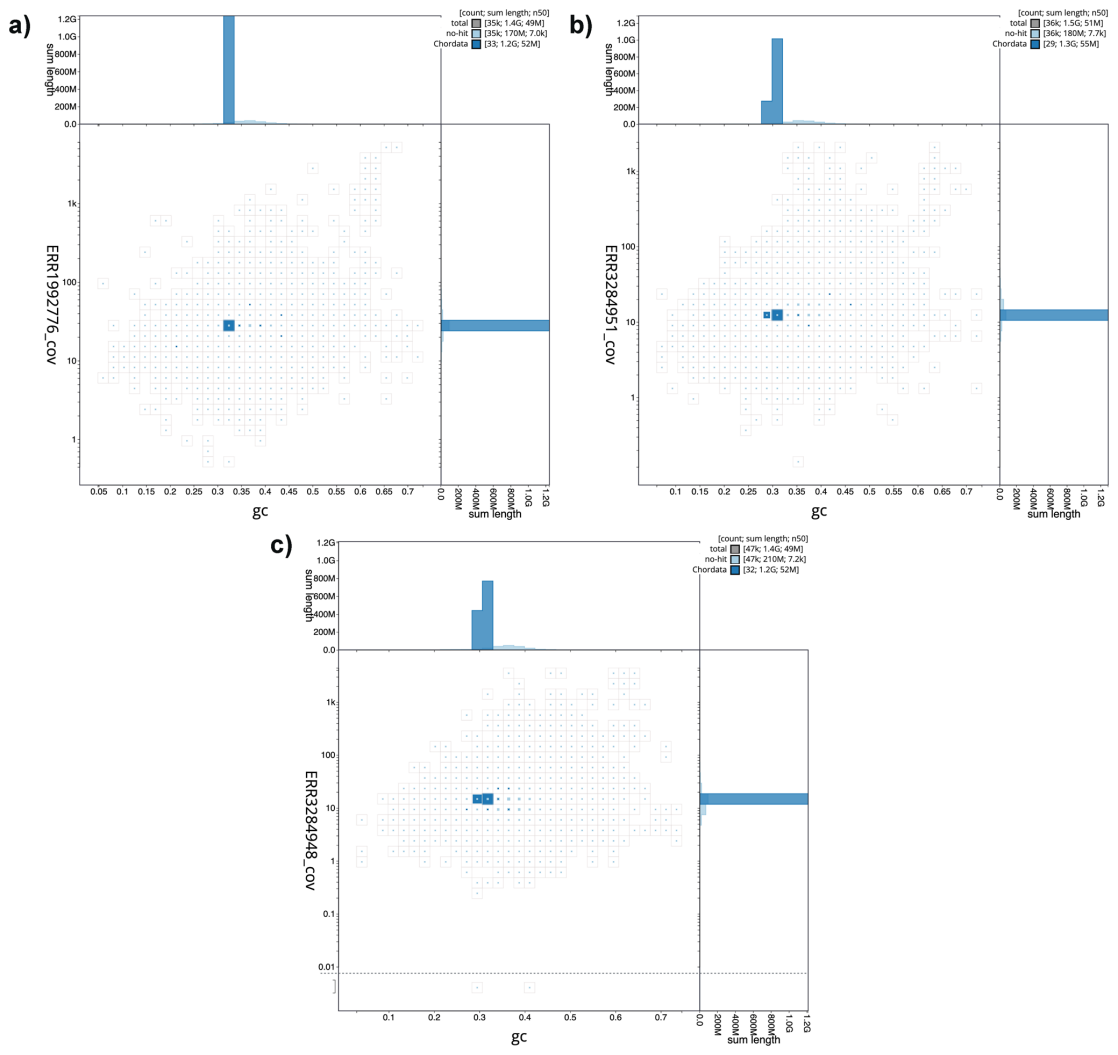
Genome assemblies of *Danio rerio* **a**) fDreABz2.1, **b**) fDreNAz3.1 and **c**) fDreCBz1.1: BlobToolKit GC-coverage plot. Scaffolds are coloured by phylum. Circles are sized in proportion to scaffold length. Histograms show the distribution of scaffold length sum along each axis. The blob plots can be viewed interactively at the following links: fDreABz2, fDreNAz3 and fDreCBz1.

**Figure 3.**
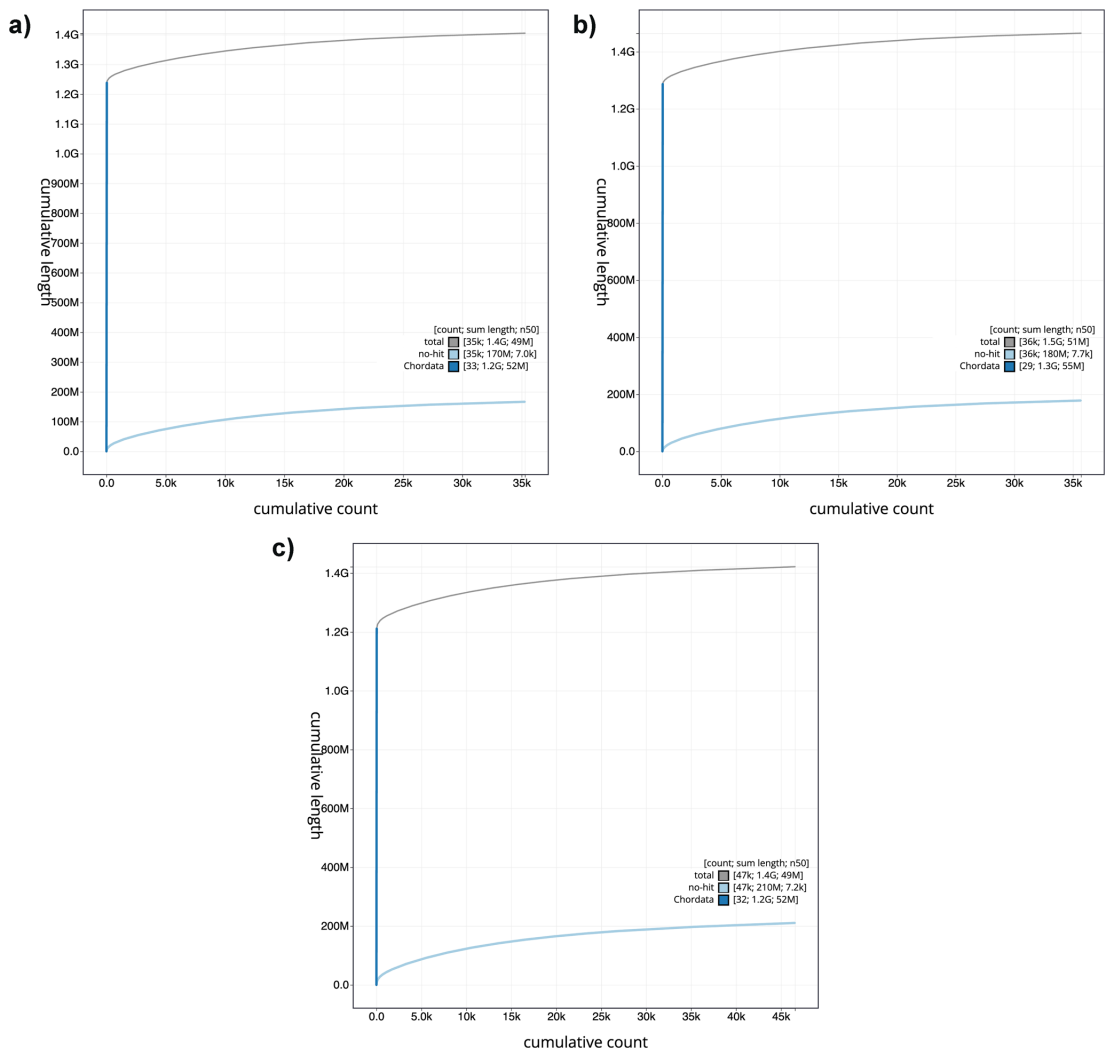
Genome assemblies of *Danio rerio* strains: BlobToolKit cumulative sequence plot **a**) fDreABz2.1, **b**) fDreNAz3.1 and **c**) fDreCBz1.1: The grey line shows cumulative length for all scaffolds. Coloured lines show cumulative lengths of scaffolds assigned to each phylum using the buscogenes taxrule. The cumulative plots can be viewed interactively at the following links: fDreABz2, fDreNAz3 and fDreCBz1.

**Figure 4.**
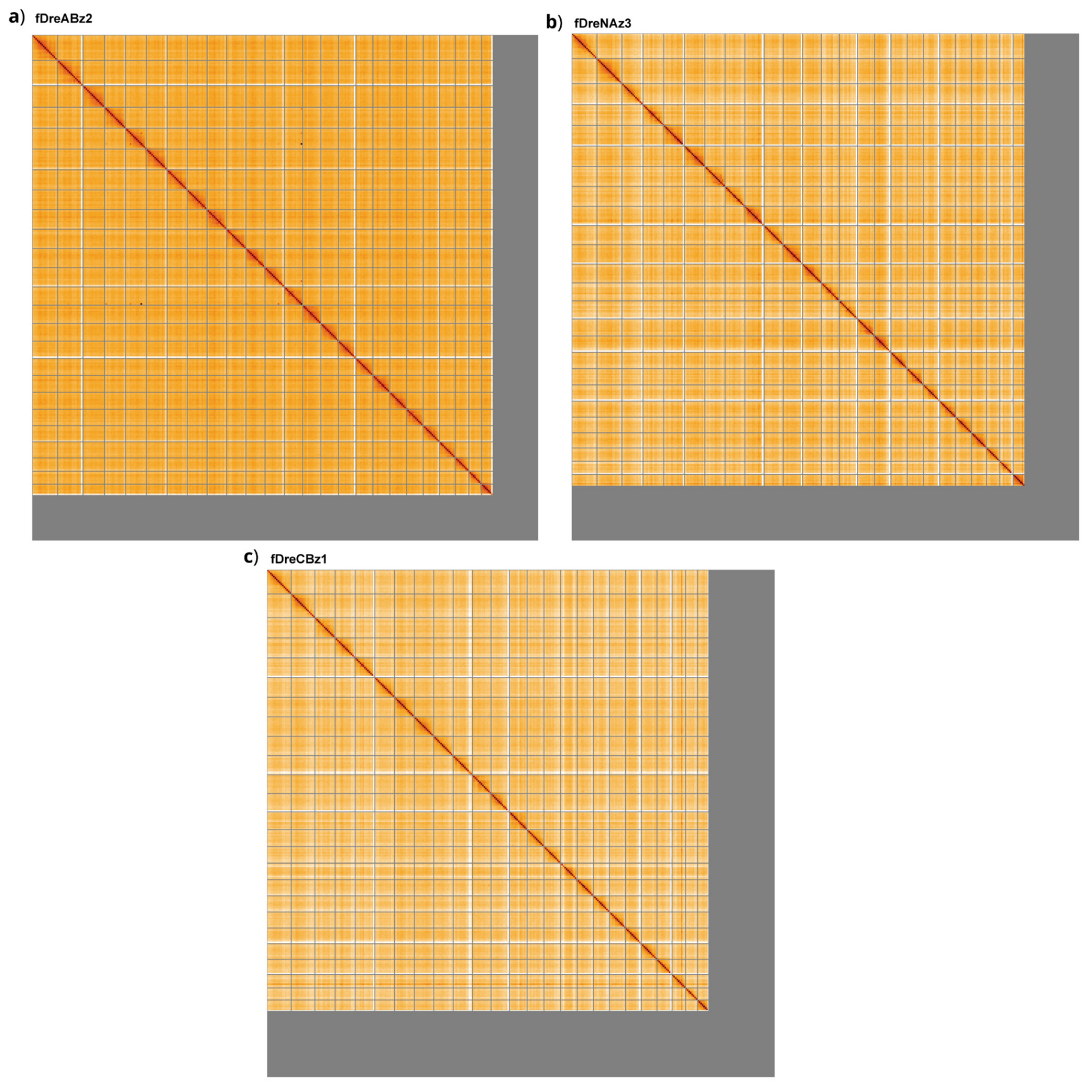
Genome assemblies of *Danio rerio* strains **a**) fDreABz2.1, **b**) fDreNAz3.1 and **c**) fDreCBz1.1: Hi-C contact map of the assemblies, visualised using HiGlass. Chromosomes are shown in order of size from left to right and top to bottom. The maps can be viewed interactively at the following links: fDreABz2, fDreNAz3 and fDreCBz1.

This project provides the genome assemblies of an individual of the *Danio rerio* AB strain (fDreABz2) based on a sample provided by Zoltan Varga at ZIRC, and of individuals of the *Danio rerio* Nadia (fDreNAz3) and Cooch Behar (fDreCBz1) strains based on samples provided by John Postlethwait, University of Oregon, via ZIRC. For each assembly, the snail plots in Figure 1 provide a summary of the assembly statistics, while the distributions of assembly scaffolds on GC proportion and coverage are shown in Figure 2. The cumulative assembly plots in Figure 3 shows curves for subsets of scaffolds assigned to different phyla. Most of the assembly sequence was assigned to 25 chromosomal-level scaffolds. Chromosome-scale scaffolds confirmed by the Hi-C data are named in order of size ( Figure 4; Table 1).

**Table 1.** Chromosomal pseudomolecules in the genome assembly of *Danio rerio*, fDreABz2.1, fDreNAz1.1 and fDreCBz1.1.

	fDreABz2.1		fDreNAz1.1		fDreCBz1.1	
Chromosome	INSDC accession	Length (Mb)	INSDC accession	Length (Mb)	INSDC accession	Length (Mb)
1	LR812594.1	56.29	LR812569.1	58.22	LR812544.1	54.09
2	LR812595.1	58.07	LR812570.1	59.28	LR812545.1	54.75
3	LR812596.1	53.15	LR812571.1	60	LR812546.1	53.43
4	LR812597.1	30.67	LR812572.1	33.44	LR812547.1	30.76
5	LR812598.1	67.56	LR812573.1	70.74	LR812548.1	66.15
6	LR812599.1	55.8	LR812574.1	58.03	LR812549.1	55.2
7	LR812600.1	68.65	LR812575.1	71.47	LR812550.1	65.28
8	LR812601.1	51.59	LR812576.1	53.62	LR812551.1	53.88
9	LR812602.1	56.26	LR812577.1	57.53	LR812552.1	54.07
10	LR812603.1	44.6	LR812578.1	46.22	LR812553.1	43.96
11	LR812604.1	45.39	LR812579.1	46.22	LR812554.1	44.34
12	LR812605.1	46.87	LR812580.1	46.63	LR812555.1	44.18
13	LR812606.1	52.21	LR812581.1	54.51	LR812556.1	52.53
14	LR812607.1	48.96	LR812582.1	51.18	LR812557.1	45.57
15	LR812608.1	45.53	LR812583.1	46.2	LR812558.1	45.53
16	LR812609.1	53.96	LR812584.1	56.18	LR812559.1	53.24
17	LR812610.1	51.58	LR812585.1	54.8	LR812560.1	50.03
18	LR812611.1	49.1	LR812586.1	51.27	LR812561.1	48.67
19	LR812612.1	47.67	LR812587.1	48.38	LR812562.1	46.31
20	LR812613.1	52.13	LR812588.1	54.69	LR812563.1	51.55
21	LR812614.1	43.49	LR812589.1	44.76	LR812564.1	42.54
22	LR812615.1	34.65	LR812590.1	37.12	LR812565.1	32.93
23	LR812616.1	45.1	LR812591.1	45.55	LR812566.1	43.62
24	LR812617.1	42.93	LR812592.1	42.69	LR812567.1	41.58
25	LR812618.1	36.09	LR812593.1	38.19	LR812568.1	36.91

For the fDreABz2.1 (GCA_903798185.1) assembly, assembly errors corrected by manual curation included 43 manual breaks, 250 joins, and 12 haplotig removals. The final assembly has a total length of 1.4 Gb in 35,278 sequence scaffolds with a scaffold N50 of 49.1 Mb ( Table 2). Most of the assembly (99.90%) was assigned to 25 chromosomal pseudomolecules. The estimated Quality Value (QV) of the final assembly is 47.4 with *k*-mer completeness of 99.95%, and the assembly has a BUSCO v5.3.2 completeness of 80.3% (single = 78.9%, duplicated = 1.4%).

**Table 2.** Genome data for *Danio rerio* strains AB, Nadia and Cooch Behar.

Project accession data			
**Assembly identifiers**	**fDreABz2.1**	**fDreNAz3.1**	**fDreCBz1.1**
**Species and strains**	*Danio rerio* AB strain	*Danio rerio* Nadia strain	*Danio rerio* Cooch Behar strain
**Specimen**	fDreABz2	fDreNAz1	fDreCBz1
**NCBI taxonomy ID**	7955		
**BioProject**	PRJEB38576	PRJEB38578	PRJEB38574
**BioSample ID**	SAMEA104026428	SAMEA104026421	SAMEA104026434
Assembly metrics*			
Consensus quality (QV)	47.4	47.3	46.4
*k*-mer completeness	79.82%	75.78%	67.59%
BUSCO **	80.3%	78.2%	76.0%
Raw data accession numbers			
10X Genomics Illumina	ERR3284942, ERR3284944, ERR3284943, ERR3284941	ERR3284950, ERR3284952, ERR3284951, ERR3284949	ERR3284942, ERR3284944, ERR3284943, ERR3284941
Genome assembly			
Assembly accession	GCA_903798185.1	GCA_903798175.1	GCA_903798165.1
Size (Mb)	1,405.10	1,465.10	1,421.80
Number of contigs	137,786	136,262	144,175
Contig N50 length (kb)	17.92 kb	17.8 kb	17.5 kb
Number of scaffolds	35,278	35,684	46,554
Scaffold N50 length (Mb)	49.10	51.3	48.7
Longest scaffold (Mb)	68.65	71.47	66.15

** BUSCO scores based on the actinopterygii_odb10 BUSCO set using v5.3.2. C = complete [S = single copy, D = duplicated], F = fragmented, M = missing, n = number of orthologues in comparison. Full sets of BUSCO scores are available for fDreABz2, fDreNAz3 and fDreCBz1.

For the fDreNAz3.1 (GCA_903798175.1) assembly, manual curation corrected the following assembly errors: 8 manual breaks, 22 joins, and removal of one haplotig. The final assembly has a total length of 1.5 Gb in 35,684 sequence scaffolds with a scaffold N50 of 51.3 Mb ( Table 2). Most ( 87.41%) of the assembly was assigned to 25 chromosomal pseudomolecules. The estimated Quality Value (QV) of the final assembly is 47.3 with *k*-mer completeness of 99.95%, and the assembly has a BUSCO v5.3.2 completeness of 78.2% (single = 76.7%, duplicated = 1.4%),

For the fDreCBz1.1 (GCA_903798165.1) assembly, 46 manual joins were made during manual curation of the assembly. The final assembly has a total length of 1.4 Gb in 46,554 sequence scaffolds with a scaffold N50 of 48.7 Mb ( Table 2). Most (84.63%) of the assembly was assigned to 25 chromosomal pseudomolecules. The estimated Quality Value (QV) of the final assembly is 46.4 with *k*-mer completeness of 99.94%, and the assembly has a BUSCO v5.3.2 completeness of 76.0 % (single = 74.5%, duplicated = 1.6%).

## Methods

### Sample acquisition and nucleic acid extraction

This project provides the genome assemblies of an individual of the *Danio rerio* AB strain based on a sample provided by Zoltan Varga at ZIRC, and of individuals of the *Danio rerio* Nadia and Cooch Behar strains based on samples provided by John Postlethwait, University of Oregon, via ZIRC.

Nucleic acid extraction was carried out using Bionano Prep Cell Culture DNA Isolation Protocol. In this method, the cells are first embedded in agarose to provide structural support during the extraction process. The agarose-embedded cells are then treated with lysis buffers to break down the cell membranes and release the DNA. The process also involves proteinase digestion to remove proteins, followed by a series of washes to purify the DNA.

### Sequencing

10X Genomics read cloud DNA sequencing libraries were constructed according to the manufacturers’ instructions. Sequencing was carried out at the Scientific Operations Core at the Wellcome Sanger Institute on an Illumina HiSeq X Ten instrument.

### Genome assembly, curation and evaluation

The assemblies fDreABz2.1 and fDreNAz3.1 are each based on 56x 10X Genomics Chromium dat, while the coverage for fDreCBz1.1 was 54x. For each of these, the assembly process included the following sequence of steps: initial assembly generation using Supernova 2.0.1 and retained haplotig identification with Purge Haplotigs. Finally, the assembly was analysed and manually improved using gEVAL ( Chow *et al.*, 2016), and marker placement from the high-density genetic map SATmap ( Howe *et al.*, 2013).

To evaluate the final genome assemblies, Hi-C maps were produced using bwa-mem2 ( Vasimuddin *et al.*, 2019) in the Cooler file format ( Abdennur & Mirny, 2020). To assess the assembly metrics, the *k*-mer completeness and QV consensus quality values were calculated in Merqury.FK ( Rhie *et al.*, 2020). Each genome was also analysed within the BlobToolKit environment ( Challis *et al.*, 2020) and BUSCO scores ( Manni *et al.*, 2021) were calculated. All BUSCO runs were done with using the actinopterygii_odb10 reference set ( *n* = 3,640).

Table 3 contains a list of relevant software tool versions and sources.

**Table 3.** Software tools: versions and sources.

Software tool	Version	Source
bcftools consensus	1.9	http://samtools.github.io/bcftools/bcftools.html
BlobToolKit	4.2.1	https://github.com/blobtoolkit/blobtoolkit
BUSCO	5.3.2	https://gitlab.com/ezlab/busco
FreeBayes	1.3.1-17-gaa2ace8	https://github.com/freebayes/freebayes
gEVAL	2016	https://geval.org.uk/
Long Ranger ALIGN	2.2.2	https://support.10xgenomics.com/genome-exome/software/pipelines/latest/advanced/other-pipelines
Merqury	1.1.2	https://github.com/thegenemyers/MERQURY.FK
minimap	2.24-r1122	https://github.com/lh3/minimap2
MitoVGP	2.2	https://github.com/gf777/mitoVGP
purge_dups	1.2.3	https://github.com/dfguan/purge_dups
scaff10x	4.2	https://github.com/wtsi-hpag/Scaff10X

### Wellcome Sanger Institute – Legal and Governance

The materials that have contributed to this genome note have been supplied by ZIRC. The Wellcome Sanger Institute employs a process whereby due diligence is carried out proportionate to the nature of the materials themselves, and the circumstances under which they have been/are to be collected and provided for use. The purpose of this is to address and mitigate any potential legal and/or ethical implications of receipt and use of the materials as part of the research project, and to ensure that in doing so we align with best practice wherever possible.

The overarching areas of consideration are:

•   Ethical review of provenance and sourcing of the material

•   Legality of collection, transfer and use (national and international)

Each transfer of samples is undertaken according to a Research Collaboration Agreement or Material Transfer Agreement entered into by the Tree of Life collaborator, Genome Research Limited (operating as the Wellcome Sanger Institute) and in some circumstances other Tree of Life collaborators.

## Data Availability

European Nucleotide Archive: *Danio rerio* AB strain (zebrafish) Accession number PRJEB38576; https://identifiers.org/ena.embl/PRJEB38576. European Nucleotide Archive: *Danio rerio* Nadia strain (zebrafish). Accession number PRJEB38578; https://identifiers.org/ena.embl/PRJEB38578. European Nucleotide Archive: *Danio rerio* Cooch Behar strain (zebrafish). Accession number PRJEB38574; https://identifiers.org/ena.embl/PRJEB38574. The assemblies are provided by the Wellcome Sanger Institute Tree of Life Programme in collaboration with Cambridge University. The data under this project are made available subject to the Tree of Life data use policy ( https://www.sanger.ac.uk/science/programmes/tree-of-life). The *Danio rerio* genome sequencing initiative is part of the Vertebrate Genomes Project. All raw sequence data and the assembly have been deposited in INSDC databases. Raw data and assembly accession identifiers are reported in Table 1.
