# Supplementary material for: The chromosome-level genome sequences of Danio rerio strains AB, Nadia and Cooch Behar
Source: Wellcome Open Res. 2025 Oct 10;10:559. [Version 1] doi: 10.12688/wellcomeopenres.25012.1 (PMC12756601; doi:10.12688/wellcomeopenres.25012.1)
Supplement: Peer review history

# Reviewer report: Reviewer response for version 1

**DOI:** 10.21956/wellcomeopenres.27555.r141555

**Published:** 2025-12-31

**Authors:** Qinggang Xue

**Affiliations:** 1 Zhejiang Wanli University, Ningbo, Zhejiang, China

The genome sequences of different zebrafish strains may facilitate the study of their unique genetic characteristics.

  

1. Clarify the terminology consistency of "chromosome-level" and "chromosome-anchored" throughout the manuscript, as the current use of "chromosome-level" may be misleading given the varying proportions of assemblies anchored to chromosomal pseudomolecules (99.90% for AB, 87.41% for Nadia, 84.63% for Cooch Behar). 
2. BUSCO assessment indicates that 12.7%, 14.6%, and 16.3% of the single-copy orthologs are missing in the three genomes, respectively. Does this imply the absence of certain sequences and insufficient completeness of the genome assemblies?
3. The Contig N50 values of the three assemblies (17.92 kb for fDreABz2.1, 17.8 kb for fDreNAz3.1, and 17.5 kb for fDreCBz1.1) appear quite low when compared to recently released chromosomal-level genome assemblies of zebrafish or closely related teleost species. Contemporary genome projects leveraging advanced sequencing technologies (e.g., PacBio HiFi long reads, Oxford Nanopore ultra-long reads) and haplotype-aware assembly tools (e.g., hifiasm, Flye) have consistently achieved Contig N50 values in the megabase (Mb) range—often exceeding 10 Mb or even 100 Mb for T2T (Telomere-to-Telomere) complete assemblies. Please discuss the relatively small Contig N50 sizes.

Are sufficient details of methods and materials provided to allow replication by others?

Yes

Is the rationale for creating the dataset(s) clearly described?

Yes

Are the datasets clearly presented in a useable and accessible format?

Partly

Are the protocols appropriate and is the work technically sound?

Partly

Reviewer Expertise:

genomics,evolution,molecular biology

I confirm that I have read this submission and believe that I have an appropriate level of expertise to confirm that it is of an acceptable scientific standard.

# Reviewer report: Reviewer response for version 1

**DOI:** 10.21956/wellcomeopenres.27555.r137604

**Published:** 2025-12-08

**Authors:** Bingpeng Xing

**Affiliations:** 1 Third Institute of Oceanography Ministry of Natural Resources, Xiamen, Fujian, China

1 The study provides assemblies and publicly accessible data for three Danio rerio strains (AB, Nadia, and Cooch Behar). However, the designation “chromosome-level” should be used more cautiously; please revise to “chromosome-anchored.”

 2“Most of the assembly is scaffolded into 25 chromosomal pseudomolecules …” “Most” is ambiguous and could be interpreted as >95% for all strains. I suggest: “For the AB strain, 99.90% of the assembly was placed into 25 chromosomes, whereas 87.41% and 84.63% of the assemblies for Nadia and Cooch Behar were anchored to 25 chromosomal pseudomolecules.”

 3“the genome of this teleost fish was fully sequenced in 2013 …”

 Please add, at the end of this sentence/paragraph, a brief statement clarifying the incremental contribution of the present work (multi-strain value) and contrasting current standards (e.g., T2T completeness). Emphasize that this study offers multi-strain resources but is not at the same level of contiguity as fully complete (T2T) assemblies, to avoid the impression that it replaces the established reference.

 4“Whilst the zebrafish reference genome assemblies have historically been based on sequencing the Tuebingen strain …”

 The background lacks up-to-date context and comparisons; please cite recent HiFi long-read assemblies and current VGP standards.

 5“We present here the genome assemblies for three zebrafish strains: AB, Nadia, and Cooch Behar.”

 Please add a sentence explaining the scientific significance (e.g., value for comparative genomics, population structure, and strain-specific variation).

 6“Most of the assembly sequence was assigned to 25 chromosomal-level scaffolds.”

 Consider revising to:

 “The majority of sequences were assigned to 25 chromosomal pseudomolecules, accounting for 99.90%, 87.41%, and 84.63% of the assemblies for AB, Nadia, and Cooch Behar, respectively.”

 7 The main text reports k-mer completeness as 99.94–99.95%, but Table 2 lists 79.82/75.78/67.59% for AB/Nadia/CB, respectively. This discrepancy should be checked and reconciled immediately.

 8“10X Genomics read cloud DNA sequencing was performed on HiSeq X Ten.”

 Please provide missing details: total yield (Gb) per strain, read length, coverage, duplication/barcode statistics, and any filtering steps.

 9“Hi-C maps were produced using bwa-mem2 and visualized with Cooler format.”

 Please specify the Hi-C library chemistry/enzyme, sequencing depth, and the software used for scaffolding/anchoring (e.g., 3D-DNA/YaHS/SALSA) with key parameters.

 10 Table 1: Please verify and standardize the Nadia entry (appears as fDreNAz1.1 in one place and fDreNAz3.1 elsewhere). Ensure consistency of version and accession.

 11 If feasible, I recommend adding PacBio HiFi data (≥25–30×) and assembling with hifiasm (haplotype/trio-aware), followed by YaHS/3D-DNA + Hi-C for anchoring, and comprehensive evaluation with Merqury/BUSCO/QUAST; consider OGM/genetic maps to validate structural consistency where appropriate.

Are sufficient details of methods and materials provided to allow replication by others?

Partly

Is the rationale for creating the dataset(s) clearly described?

Yes

Are the datasets clearly presented in a useable and accessible format?

Yes

Are the protocols appropriate and is the work technically sound?

Partly

Reviewer Expertise:

biodiversity，genome，molecular biology，molecular ecology

I confirm that I have read this submission and believe that I have an appropriate level of expertise to confirm that it is of an acceptable scientific standard, however I have significant reservations, as outlined above.

# Reviewer report: Reviewer response for version 1

**DOI:** 10.21956/wellcomeopenres.27555.r136023

**Published:** 2025-11-10

**Authors:** Karim Gharbi

**Affiliations:** 1 Earlham Institute, Norwich, England, UK

This data note describes genome assemblies for three zebrafish laboratory strains. The assemblies were publicly released in 2020, with the report serving as a retrospective record. The note includes a high-level overview of the species as a model organism for biological research followed by a standardised genome report. Assembly statistics are consistent with the input data types (10X linked-reads, HiC), including a large number of scaffolds. Overall, the study and associated assemblies represent a major contribution to biomedical research. 

**Main comment**

 - the methods used to generate HiC data for each strain seem to be missing

**Minor comments**

 - this sentence is repeated three times within the report, which seems excessive: 'This project provides the genome assemblies of an individual of the *Danio rerio* AB strain based on a sample provided by Zoltan Varga at ZIRC, and of individuals of the *Danio rerio* Nadia and Cooch Behar strains based on samples provided by John Postlethwait, University of Oregon, via ZIRC'

 -  the unit (kb) in the Contig N50 length values ics for each strain is redundant as it is defined in the header

Are sufficient details of methods and materials provided to allow replication by others?

Partly

Is the rationale for creating the dataset(s) clearly described?

Yes

Are the datasets clearly presented in a useable and accessible format?

Yes

Are the protocols appropriate and is the work technically sound?

Yes

Reviewer Expertise:

Genomics, bioinformatics

I confirm that I have read this submission and believe that I have an appropriate level of expertise to confirm that it is of an acceptable scientific standard, however I have significant reservations, as outlined above.
